# Outcomes and return to sport after osteochondral autograft transplantation for osteochondritis dissecans of the capitellum: a systematic review

**DOI:** 10.1016/j.xrrt.2024.02.011

**Published:** 2024-03-28

**Authors:** Gabriel Lane, Matthew V. Smith, Charles A. Goldfarb, Rogelio A. Coronado, Eric N. Bowman

**Affiliations:** a Meharry Medical College, Nashville, TN, USA; b Washington University in St. Louis, Chesterfield, MO, USA; c Division of Hand and Microsurgery, Washington University in St. Louis, Chesterfield, MO, USA; d Department of Orthopaedics, Vanderbilt University Medical Center, Nashville, TN, USA

**Keywords:** Osteochondral autograft transplantation, OAT, Osteochondritis dissecans, OCD, Capitellum, Elbow, Baseball, Return to sport

## Abstract

**Background:**

Capitellar osteochondritis dissecans (OCD) lesions are common in athletes. Osteochondral autograft transfer (OAT) is one possible treatment option, though outcomes including return to sport (RTS) data are limited to small series. The purpose of this study was to systematically review RTS following OAT for capitellar OCD lesions. Our secondary objectives were to evaluate patient-reported outcomes (PROs), range of motion (ROM), and complications after OAT.

**Methods:**

PubMed, Embase, and Cumulative Index to Nursing and Allied Health Literature were searched for peer-reviewed articles on “osteochondral autograft transfer” and related terms for capitellar OCD lesions. Articles were included if they reported an RTS rate and had a follow-up time point of at least 12 months. Data on RTS rates, PRO measures, complications, and ROM were extracted. Articles were assessed for methodological quality using the Methodological Index for Non-randomized Studies criteria.

**Results:**

Six hundred sixty-six articles were retrieved, and 24 articles (470 patients) met the inclusion criteria. In total, 454/470 patients (97%) returned to sports following OAT for OCD. The RTS rate ranged from 79% to 100%. Return to previous level of performance ranged from 10% to 100%. Timmerman-Andrews postoperative scores (range = 169-193) were most often reported, with 87% of patients showing scores within the excellent range. Disabilities of the Arm, Shoulder, and Hand and Japanese Orthopedic Association scores were also excellent postoperatively for all studies reporting, with higher scores among centralized lesions vs. lateral.

**Conclusions:**

Following OAT for capitellar OCD lesions, RTS rates are high; however, athletes should be counseled on the potential of a return to lower performance or the need to change positions. Lateral lesion location may negatively impact outcomes. PRO scores are typically excellent and postoperative ROM consistently improves. This information helps counsel patients regarding expectations and outcomes of OAT for OCD of the capitellum.

Osteochondritis dissecans (OCD) of the elbow is common among youth baseball players and gymnasts, with an overall incidence of 6.0 per 100,000 in the United States, with a 1.8% cumulative incidence per year among youth baseball players.^9^^,^^17^^,^^36^ Capitellar OCD may present as an incidental finding on imaging with only 35% of patients with capitellar OCD reporting pain.^17^ Patients presenting with symptoms of pain or mechanical symptoms impairing athletic performance are treated based on patient age and stability of the lesion. The goal of treatment is to restore full, painless range of motion (ROM) in order to return the athlete to his or her previous level of performance. Nonoperative treatment is often successful in stable lesions, particularly those with an open capitellar physis.^31^ When conservative treatments for all-sized lesions fail and with large or unstable lesions, surgical management may be beneficial.

Surgical options for OCD include removal of loose bodies, drilling, débridement, microfracture, chondrocyte implantation, bone-peg grafting, and osteochondral transplants.^30^ Osteochondral autograft transfer (OAT) is a cartilage restoration procedure in which a cartilage and bone plug is harvested, often from a non-weight-bearing aspect of the distal femoral condyle, then transferred into the defect.^24^ While outcomes have generally been described as favorable with limited donor-site morbidity, case series are limited, and several recent studies have been published since previous systematic reviews. The purpose of this study was to systematically evaluate the literature regarding OAT return to sport (RTS), patient-reported outcomes (PROs), and ROM.

## Methods

### Study design

This study is a systematic review of published peer-reviewed studies reporting RTS, PROs, ROM, and complications after OAT for OCD. The design and conduct of this systematic review followed guidelines from the Cochrane Handbook for Systematic Reviews. Reporting of this review followed the Preferred Reporting Items for Systematic Reviews and Meta-Analysis (PRISMA) guidelines.^23^ Meta-analyses were not performed as included articles are level III and IV evidence studies.

### Literature search

**Figure 1:**
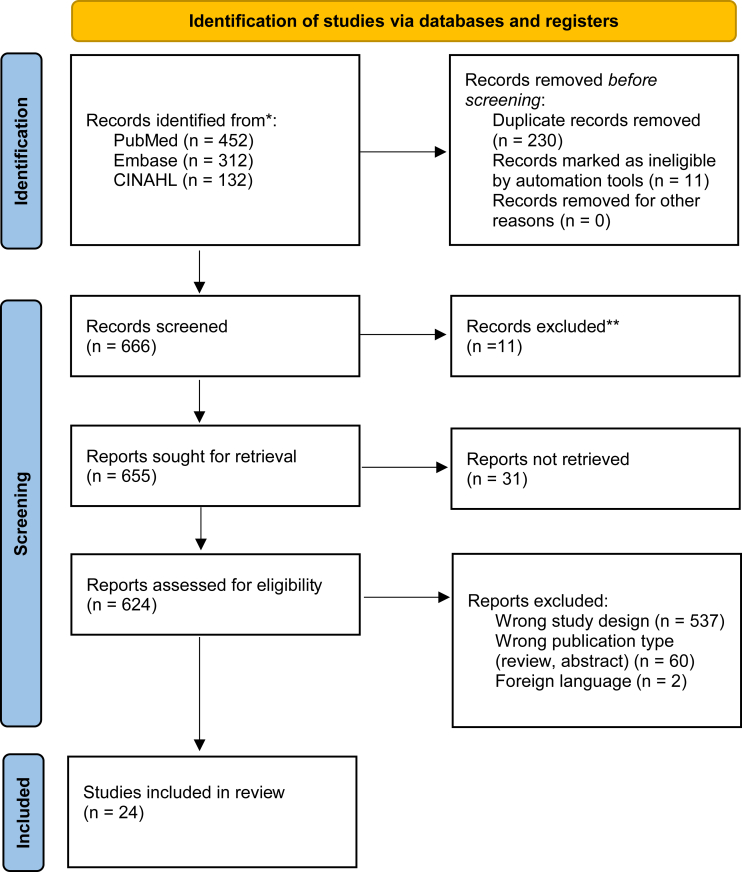
PRISMA flow chart. *PRISMA*, Preferred Reporting Items for Systematic Reviews and Meta-Analysis.

A comprehensive literature search was conducted in July 2022 to evaluate the literature from inception. Embase and Cumulative Index to Nursing and Allied Health Literature databases were searched using a prespecified search strategy (Fig. 1). Related terms for osteochondral autograft transplantation, grafting, elbow, capitellum, and outcomes were combined and searched (the complete list of Boolean terms is found in Appendix 1). The resulting articles were exported into the EndNote reference management tool (Clarivate, Philadelphia, PA, USA) for records management.^32^

### Selection criteria

All articles were independently reviewed by two authors (E.N.B. and G.C.L.) for inclusion, with a third reviewer available to resolve any disagreements. The online software Rayyan was used to review and select articles.^20^ Peer-reviewed primary research articles in the English language regarding capitellar OCD treatment with OAT or stratified for OAT with follow-up for at least 12 months were included. We required that articles include a quantitative report of (RTS) and at least one PRO measure or ROM measurement.

Articles were excluded if they were not available in the English language, were not primary research articles (including book chapters, systematic reviews, editorial commentaries, narrative reviews, animal, or cadaver studies), were surgical technique papers which did not include outcome measures, or if they were reports that did not include outcomes pertaining to OAT (fixation, débridement, allograft transplantation without OAT data). Twenty-four articles were included.

### Data analysis

The following data were extracted: authors and year published, patient demographics (number of elbows, age, sex, skeletal maturity, level of play), patient characteristics (dominant arm involvement, lesion classification, mean follow-up), outcomes (RTS rate, PRO measures, ROM, and number of revision procedures), and complications. The primary outcome measure of this study was the RTS rate regarding OATs. Preoperation and postoperation data were collected for ROM and PROs when available. Two articles were included which did not include preoperative and postoperative PROs, but these were included as information was complete for all other desired data points.^1^^,^^3^

Osteochondritis lesions were described predominantly using the International Cartilage Regeneration and Joint Preservation Society grading system.^18^ Grade 0 lesions are normal cartilage with no magnetic resonance imaging signal changes. Grade 4 lesions are the most severe and contain a loose body within the lesion or joint space. Grade 2, 3, and 4 lesions were reported among the included studies.

Outcome measures were recorded for each study. The Timmerman-Andrews (TA) scoring system^33^ defines an excellent overall score as 180-200 while a poor score is defined as less than 120. Total scores are a sum of a subjective component (patient’s perception of the following: pain, swelling, locking/catching, and difficulty performing activities) and an objective component (flexion contracture, pronation/supination, and sagittal arc of motion). Outcome measures were also reported using the Broberg and Morrey rating system, Disabilities of the Arm, Shoulder, and Hand (DASH) score, Japanese Orthopedic Association (JOA) score, and Mayo Elbow score. Higher scores are associated with better elbow function except for the DASH score, in which a lower score is associated with better function. RTS was defined as the patient being able to return to any level of competition for their primary sport. The rate at which patients returned to a lower level of sport was also extracted when included.

### Methodological quality

Methodological quality was assessed using the Methodological Index for Non-randomized Studies scoring system (Appendix 2).^29^ The Methodological Index for Non-randomized Studies instrument contains 8 items for noncomparative studies and 12 items for comparative studies to assess the risk of bias. Higher scores are correlated with a lower risk of study bias. Each study was independently reviewed by two authors (E.N.B.) and (G.C.L.) with a third author available to resolve any disagreements.

## Results

### Study selection, demographics, and lesion characteristics

The initial search yielded 452 results from PubMed, 312 results from Embase, and 132 results from Cumulative Index to Nursing and Allied Health Literature. The reference lists of articles were reviewed for missed articles and one additional article was included. After duplicates were removed, a total of 666 unique articles were identified for review with 24 meeting inclusion criteria. The summary of these studies, demographic, and lesion characteristics are shown in Table I. Included studies were from 2005 to 2021. There were 3 Level III cohort studies^16^^,^^35^^,^^39^ 22 Level IV studies. Overall, 470 patients were included. The age of patients ranged from 13 to 22 years of age. Five studies reported the skeletal maturity of patients.^6^^,^^14^^,^^15^^,^^26^^,^^28^ All except 3 studies had a patient population strictly composed of athletes.^3^^,^^27^^,^^37^ Eleven studies noted whether the lesion was in the dominant arm^1^, ^2^, ^3^^,^^8^^,^^15^^,^^16^^,^^18^^,^^19^^,^^26^^,^^34^^,^^37^; 7 studies reported that only the dominant arm was involved.^1^^,^^8^^,^^15^^,^^16^^,^^18^^,^^19^^,^^26^^,^^34^

**Table I:** Demographic and lesion characteristics reported in 24 included studies.

Study	Level of evidence	Number of patients	Mean patient age	Male: Female	Proportion skeletally mature	Athletes only?	Proportion with dominant arm involvement	ICRS lesion grade
Allagui et al^1^ (2012)	IV	1	17	1:0	NR	Yes	1	3
Ansah et al^2^ (2007)	IV	5	17	4:3∗	NR	Yes	0.2	4
Ayzenberg et al^3^ (2021)	IV	15	13.7	11:4	NR	No	0.6	4
Bilsel et al^5^ (2010)	IV	1	17	1:0	NR	Yes	NR	3
Funakoshi et al^6^ (2018)	IV	22	13.5	22:0	NR	Yes	NR	2,3,4
Iwasaki et al^8^ (2006)	IV	8	14	8:0	NR	Yes	1	3,4
Iwasaki et al^7^ (2009)	IV	19	14.2	19:0	1	Yes	NR	3,4
Lyons et al^13^ (2015)	IV	11	14.5	10:1	NR	Yes	NR	NR
Maniwa et al^14^ (2017)	IV	9	13.7	9:0	0.36	Yes	NR	NR
Maruyama et al^15^ (2014)	IV	33	13.6	33:0	0.88	Yes	1	3,4
Matsuura et al^17^ (2017)	III	87	13.2	86:1	NR	Yes	1	3,4
Mihara et al^18^ (2010)	IV	7	13.3	7:0	NR	Yes	1	3,4
Mirzayan et al^19^ (2016)	IV	9	15.3	9:0	NR	Yes	1	2,3,4
Nishinaka et al^21^ (2014)	IV	22	13.9	22:0	NR	Yes	NR	3,4
Ovesen et al^22^ (2011)	IV	10	18	4:6	NR	Yes	NR	NR
Pederzini et al^25^ (2017)	IV	9	22.4	7:2	NR	Yes	NR	NR
Sato et al^26^ (2018)	IV	72	14.3	71:1	0.972	Yes	1	3,4
Shimada et al^27^ (2012)	IV	26	16	26:0	NR	No	NR	3,4
Shimada et al^28^ (2005)	IV	10	14.3	10:0	0.5	Yes	NR	NR
Tsuda et al^34^ (2005)	IV	3	12.7	2:1	NA	Yes	1	NR
Ueda et al^35^ (2021)	III	29	14	29:0	NA	Yes	NR	NR
Weigelt et al^37^ (2015)	IV	14	18	9:5	NA	No	0.57	3,4
Yamagami et al^39^ (2018)	III	30	14	30:0	NA	Yes	NR	NR
Yamamoto et al^40^ (2006)	IV	18	13.6	18:0	NA	Yes	NR	3,4

*ICRS*, International Cartilage Repair Society; *NR*, not reported.

∗ Ratio reports entire study population.

### Return to sport

Outcome scores for the included studies are summarized in Table II. RTS was achieved in 454/470 patients (97%). RTS rate ranged from 79% to 100%; all but 5 studies reported a RTS rate of 100%. Seven studies reported a proportion of their study population, 38 patients in total, returning to a reduced level of sport (range: 10%-100%).^6^^,^^8^^,^^12^^,^^15^^,^^21^^,^^28^^,^^40^ Baseball players, and pitchers specifically, were more likely to RTS at a lower level of play or not able to return at all. Time to RTS among included articles ranged from 4 months to 12 months.

**Table II:** Summary findings for return to sport rates and patient outcomes.

Article	Mean follow-up (months)	RTS rate (%)	Return to reduced level of sport (%)	Preop patient-reported outcome	Postop patient-reported outcome	Preop range of motion	Postop range of motion	Number of revisions/2nd looks
Allagui et al^1^ 2012	24	100	NR	NR	NR	−15° extension, 130° flexion, 115° total arc	NR	0
Ansah et al^2^ 2007	59.3	100	NR	B&M; 76.3	B&M; 97.6	4.7° extension lag, 12.9° flexion lag	0 °extension lag, 0° flexion lag	0
Ayzenberg et al^3^ 2021	29.5	100	NR	NR	NR	121.9° (arc), 131.2° (passive flexion), 9.2° flexion contracture	139.1° (arc), 142.7° (passive flexion), 2.7° flexion contracture	2
Bilsel et al^5^ 2010	40	100	NR	DASH; 12	DASH; 1	5° extension, 120° flexion	0 ° extension, 140° flexion	0
Funakoshi et al^6^ 2018	27.5	100	90	T-A; 125 ± 30.1 (central lesion) T-A; 138.3 ± 34.5 (lateral lesion)	T-A; 193.5 ± 11.3 (central lesion) T-A; 186.7 ± 18.1 (lateral lesion)	NR	132.7° (123°-142.4°) total arc	0
Iwasaki et al^8^ 2006	24	100	25	T-A; 134	T-A; 183	110° (93°-127°) total arc	122° (108°-136°) total arc	0
Iwasaki et al^7^ 2009	45.1	100	NR	T-A; 131 (IQR 108-154)	T-A; 191 (IQR 176-206)	112° (IQR 95°-129°) total arc	128° (IQR 116°-140°) total arc	0
Lyons et al^13^ 2015	22.7	100	0	NR	DASH; 1.4 (0.6-2.1)	126° flexion, 21° extension	141° flexion, 5° extension	1
Maniwa et al^14^ 2017	21.1	100	NR	JOA; 68 (IQR 51.8-84.2)	JOA; 98.7 (IQR 96-101.4)	NR	NR	NR
Maruyama et al^15^ 2014	28.4	96	6	T-A; 143	T-A; 190	116° (IQR 95°-137°) total arc, −13° (IQR -27° to +1°) extension, 130° (IQR 110°-140°) flexion	133° (IQR 116°-150°) total arc, −13° (IQR -17° to −11°) extension, 136° (IQR 127°-145°) flexion	0
Matsuura et al^16^ 2017	43.4	93	NR	T-A; 139.7 ± 38.8 (central lesion) T-A; 136.7 ± 36.8 (lateral lesion)	T-A; 193.8 ± 9.5 (central lesion) T-A; 185.3 ± 17.6 (lateral lesion)	Central lesion: −7.0° ± 16.8 extension, 132.2° ± 10.8 flexion Lateral lesion: −8.43° ± 10.8 extension, 130.7° ± 9.3	Central lesion: 2.3° ± 5.4 extension, 138.6° ± 6.9 flexion, Lateral lesion: −3.2° ± 8.7 extension, 135.7° ± 7.0 flexion	NR
Mihara et al^18^ 2010	37.4	100	NR	T-A; 161.4	T-A; 185	100.7° (87.17°-127.17°) total arc, −12.5° (−24.5° to −0.5°) extension, 119.6° (106.8°-132.4°) flexion	117.9° (100°-135.6°) total arc, −7.3° (−17.7° to 3.1°) extension, 126.2° (116.7° - 135.7°) flexion	0
Mirzayan et al^19^ 2016	48.4	100	NR	DASH; 35.2	DASH; 5.4	32° lack of extension	6° lack of extension	2
Nishinaka et al^21^ 2014	27	100	41	T-A; 121.9	T-A; 169	99° total arc, 118° flexion, −19° extension	113° total arc, 124° flexion, −11° extension	4
Ovesen et al^22^ 2011	30	100	NR	Mayo Elbow Score; 71 (55-85)	Mayo Elbow Score; 93.5 (85-100)	128° total arc, 137° (120°-140°) flexion, −9.5° (−25° to 0°) extension	136.5° total arc, 138.5° (125°-140°) flexion, −2° (−10° to 0°) extension	0
Pederzini et al^25^ 2017	48	100	NR	DASH; 44.5	DASH; 2.50	19.3° (10°-30°) extension, 128° (120°-140°) flexion	1.2° (0°-3°) extension, 138.6° (130°-145°) flexion	0
Sato et al^26^ 2018	57	100	NR	T-A; 101	T-A; 190	101° total arc, −21° extension, 122° flexion	132° total arc, −4° extension, 136° flexion	2
Shimada et al^27^ 2012	36	100	NR	T-A; 111	T-A; 190	110° total arc, 126° flexion, 16° extension	130° total arc, 133° flexion, −3° extension	5
Shimada et al^28^ 2005	25.5	100	38	JOA; 80.6	JOA; 93.8	NR	NR	6
Tsuda et al^34^ 2005	16.3	100	NR	NR	T-A; 193	130° total arc, 131.7° flexion, −1.7° extension	NR	NR
Ueda et al^35^ 2021	84	100	NR	T-A; 126	T-A; 175	112° total arc, 130° flexion, −18° extension	127° total arc, 133° flexion, −6° extension	NR
Weigelt et al^37^ 2015	84	79	NR	NR	B&M; 95.1	NR	133° total arc	NR
Yamagami et al^39^ 2018	36	90	NR	JOA; 74.5	JOA; 99.2	Central lesion: 130° flexion, −9° extension Lateral localized lesion: 117° flexion, −17° extension Lateral widespread lesion: 128° flexion, −10° extension	Central lesion: 140° flexion, −2° extension Lateral localized lesion: 137° flexion, −1° extension Lateral widespread lesion: 133° flexion, −6° extension	4
Yamamoto et al^40^ 2006	45	89	11	T-A; 150	T-A; 181	114° total arc	126° total arc	1

*B&M*, Broberg-Morrey score; *DASH*, Disabilities of the Arm, Shoulder, and Hand score; *IQR*, interquartile range; *JOA*, Japanese Orthopedic Association; *NR*; Not Reported; *T-A*, Timmerman-Andrews score.

### Patient-reported outcome measures

The mean measurements of function improved postoperatively in all studies. Thirteen studies utilized the TA scoring system; 87% of patients had excellent outcomes with a mean score of 186.4 (range: 169-193.8). Four studies reported outcomes using the DASH scoring system with a mean score of 2.92 (range: 5.4-1.0). Three studies (37 patients) utilizing the JOA scoring system reported a mean score of 97.6 (range: 93.8-99.2). In studies stratifying for lesion location, Matsuura reported significantly higher TA scores at final follow-up for central lesions (193.8 ± 9.5) vs. lateral lesions (185.3 ± 17.6).^16^ Yamagami reported a significant increase in JOA scores preoperatively to postoperatively in the central (74.5 to 99.2), lateral localized (63.7 to 95.4), and lateral widespread (75.9 to 90.5) cohorts.^39^ Funakoshi reported similar results with significant TA improvements preoperatively to postoperatively in both central (125 to 193.5) and lateral (138.3 to 186.7) lesion cohorts.^6^

### Range of motion

ROM was reported in 22 of the 24 articles as degrees of total arc, flexion, extension, extension lag, or flexion lag. Preoperative extension data were available for 406 patients and preoperative flexion data were available for 401 patients. These patients had a respective average of 9.1° lack of full extension and 126.8° of flexion preoperatively. Postoperative extension data were available for 416 patients and postoperative flexion data were available for 411 patients. These patients had a respective average of 3.6° lack of full extension and 135.6° of flexion postoperatively. Two studies stratified for lesion location. Matsuura reported significantly better extension postoperatively in central lesion patients vs lateral lesion patients.^16^ Yamagami reported a significant improvement in ROM preoperatively to postoperatively in both the central lesion and lateral location lesion cohorts.^39^

### Revisions and second looks

All but five studies reported second looks and revisions. The number of revision and second look procedures ranged from 0 to 6 per study. Ayzenberg et al reported two revision procedures in which a back-fill cyst and the removal of a loose body necessitated a second look. Lyons et al reported a single postoperative complication in which irrigation and débridement of a superficial wound infection were performed. Mirzayan et al noted two second-look procedures: one for a flexion contracture and another for areas of fissuring adjacent to the transplanted cartilage. Nishinaka et al reported four revision procedures; two for loose bodies impairing ROM, one spur impairing ROM, and one for screw deviation which avulsed articular cartilage. Sato et al noted two second-look procedures due to the cartilage layer peeling from the graft. Both patients presented with locking symptoms after returning to sport. Shimada et al (2012) noted additional surgery in five patients due to “minor symptoms or for removal of instrumentation.” Shimada et al (2005) reported six reoperations but did not expand upon why reoperations were necessary. Yamagami et al reported four reoperations: one for resection of a loose body, and three for synovectomy or osteochondral plug transplantation. Yamamoto et al reported a single reoperation for the removal of a loose body.

### Complications/donor site morbidity

Donor site morbidity was described in 14 of the included articles and affected 26 patients (Table III). Complications of donor site morbidity varied widely across studies ranging from 0% to 62.5% of subjects. Iwasaka et al reported 5 patients (62.5%) who experienced knee joint effusions which lasted a mean of 5 weeks postoperatively.^8^ The most common complication of the autograft donor site was anterior knee pain with several participants also reporting knee pain exacerbated by high-impact exercise and heavy lifting. Three articles included in our study utilized costal cartilage as an autograft site. One patient experienced a pneumothorax as a complication of harvesting which resolved following chest tube insertion.

**Table III:** Donor site morbidity.

Article	Donor site	Discussed donor site morbidity?	Proportion of patients affected	Description of morbidity
Allagui et al^1^ 2012	Lateral femoral condyle	No	NR	NR
Ansah et al^2^ 2007	Lateral femoral condyle	Yes	20.0% (1)	Pain with high-impact loads to knee
Ayzenberg et al^3^ 2021	Lateral femoral condyle	Yes	0	NR
Bilsel et al^5^ 2010	Lateral femoral condyle	No	NR	NR
Funakoshi et al^6^ 2018	Lateral femoral condyle	Yes	9.09% (2)	Mild swelling that occurred after vigorous activities
Iwasaki et al^8^ 2006	Lateral femoral condyle	Yes	62.5% (5)	Joint effusion
Iwasaki et al^7^ 2009	Lateral femoral condyle	Yes	5.26% (1)	Mild anterior knee pain with stair-climbing
Lyons et al^13^ 2015	Lateral femoral condyle	Yes	0	NR
Maniwa et al^14^ 2017	Lateral femoral condyle	No	NR	NR
Maruyama et al^15^ 2014	Lateral femoral condyle	Yes	3.03% (1)	Mild anterior knee pain during exercise
Matsuura et al^16^ 2017	Lateral femoral condyle	No	NR	NR
Mihara et al^18^ 2010	Lateral femoral condyle	No	NR	NR
Mirzayan et al^19^ 2016	Lateral femoral condyle	No	NR	NR
Nishinaka et al^21^ 2014	Costal cartilage	No	NR	NR
Ovesen et al^22^ 2011	Lateral femoral condyle	Yes	0	NR
Pederzini et al^25^ 2017	Lateral femoral condyle	Yes	0	NR
Sato et al^26^ 2018	Costal cartilage	Yes	0	NR
Shimada et al^27^ 2012	Costal cartilage	Yes	3.85% (1)	Pneumothorax
Shimada et al^28^ 2005	Lateral femoral condyle	No	NR	NR
Tsuda et al^34^ 2005	Lateral femoral condyle	No	NR	NR
Ueda et al^35^ 2021	Lateral femoral condyle	No	NR	NR
Weigelt et al^37^ 2015	Lateral femoral condyle	Yes	57.1% (8)	Seven patients reported occasional pain during heavy lifting. One patient complained about intermittent locking sensations
Yamagami et al^39^ 2018	Lateral femoral condyle	Yes	23.3% (7)	Seven patients required needle aspiration because of postoperative intra-articular hematoma
Yamamoto et al^40^ 2006	Lateral femoral condyle	Yes	0	NR

*NR*, not reported.

## Discussion

This systematic review demonstrated that OAT for elbow OCD consistently resulted in an excellent rate of RTS and PRO measures. ROM consistently improved, and PROs were excellent in 80%-90% of patients. The only inconsistent finding was the return to previous level of performance (10%-100%), which may be impacted by sport and position played (pitchers).

PROs improved preoperatively to postoperatively across all studies. All studies, apart from Nishinaka et al and Ueda et al, reported postoperative TA scores in the excellent range. In studies stratifying for the location of the OCD lesion, patients with central lesions reported better postoperative outcomes on the TA scale compared to patients with lateral lesions. These findings are consistent with previous work by Logli et al which also reported more favorable outcomes for patients with central lesions.^11^

Our study found the RTS range among the represented studies was 79%-100% including 498 patients, with all but 5 included studies reporting a RTS rate of 100%. RTS rates in this study are consistent with prior systematic reviews of OAT for OCD. Westerman et al found a RTS rate of 95% in 164 athletes and a return to previous level of play in 94%.^38^ Average time to RTS was 6 months. Our study found a time to RTS ranging from 4 months to 12 months. Kirsch et al reported a return to play rate of 94% including patients with a mean time of 5.6 months.^10^

The majority of studies contained an athlete-only population, predominately baseball players. Systematic review by Logli et al reported a return to previous level of performance ranging from 62% to 100% with a range of return to a reduced level of 7%-41%.^11^ Pitchers had a lower return to the same level, likely due to a higher lateral compressive force when throwing. Several included studies within this systematic review reported considerable rates of return to a reduced level of sport ranging from 0% to 90%. It is important to note that two studies with RTS rates less than 100% report players leaving sports for reasons unrelated to elbow injury.^37^^,^^40^ Weigelt et al report 14% of patients and Yamamoto et al report 6% of patients leaving for reasons unrelated to elbow injury. Potential return to a lower level of sport or the need to change positions should be taken into consideration for athletes.

Complication rates were overall low, but donor site morbidity was reported ranging from 0% to 62.5% of patients across studies. Anterior knee pain was the most frequent reported complication with respect to lateral femoral condyle autograft harvesting. Our rates of donor site complications align with a prior systematic review on donor site morbidity by Bexkens et al.^4^ They postulated that the size of the autograft may increase the complication rate, as some grafts ranged up to 11 mm in diameter. They also noted that the donor site morbidity rates were similar with respect to knee and costal harvesting. The morbidity of costal autograft harvesting could be argued as more significant given the approximation of costal cartilage to the pleura and the potential for pneumothorax. The surgeon and patient should discuss possible morbidity when autograft harvesting is considered.

### Limitations

The primary limitation of this study is that it is comprised of level of evidence III and IV studies. Therefore, bias will be present due to differing practice patterns and possible selection bias. Additionally, surgical indications may vary between studies which can limit comparisons. Another limitation is the variability in reported outcome measures and follow-up. Additional higher level of evidence studies, including comparative treatment studies, are needed to provide more definitive conclusions.

## Conclusions

Following OAT for capitellar OCD lesions, RTS rates are high; however, athletes should be counseled on the potential of a return to lower performance or the need to change positions. Lateral lesion location may negatively impact outcomes. PRO scores are typically excellent and postoperative ROM consistently improves. This information helps counsel patients regarding expectations and outcomes of OAT for OCD of the capitellum

## Disclaimers:

Funding: Dr. Coronado was supported by a Vanderbilt Clinical and Translational Research Scholars award (NIH/NCATS KL2TR002245) during the time of this manuscript.

Conflicts of interest: The authors, their immediate families, and any research foundation with which they are affiliated have not received any financial payments or other benefits from any commercial entity related to the subject of this article.
