# Supplementary material for: Outcomes and return to sport after osteochondral autograft transplantation for osteochondritis dissecans of the capitellum: a systematic review
Source: JSES Rev Rep Tech. 2024 Mar 28;4(3):563–70. doi: 10.1016/j.xrrt.2024.02.011 (PMC11329020; doi:10.1016/j.xrrt.2024.02.011)
Supplement: Appendix [file mmc1.docx]

**Appendix**

**Appendix 1. Search Strategy**

Boolean logic was used to create the following search structure: [("adolescent") OR (adolescent*) OR ("minors") OR (minor*) OR ("Child") OR (child*)) OR (pediatric*) OR (young*) OR (youth)] AND [("osteochondritis") OR ("osteochondritis dissecans") OR ("osteochondritis dissecans") OR (osteochondritis)] AND [("elbow joint") OR ("elbow" OR (elbow joint)) OR (elbow) OR ("Humerus") OR (capitellum) OR (humerus) OR ("upper extremity") OR ("upper extremity")) AND (("allografts") OR ("autografts") OR (allograft OR (autograft) OR (osteochondral) OR (surgery) OR (surgical procedures) OR ("transplantation" OR ("transplants") OR (operative) OR ("arthroscopy") OR ("orthopedic procedures") OR (arthroscopy) OR ("orthopedic procedures" OR ("surgical procedures") OR (transplantation) OR (transplants)] AND [("return to sport") OR ("treatment outcome") OR ("sports") OR ("range of motion, articular") OR ("disability evaluation") OR ("recovery of function") OR ("pain") OR ("health status") OR (sport*) OR (outcome*) OR (motion) OR (disability) OR (function*) OR (health) OR (pain) OR (play) OR (activity)].

| **Appendix 2.** MINORS Scoring (n=25) | | |
| --- | --- | --- |
| **Article** | **MINORS Score** | **MINORS %** |
| Allagui et al.[^24^](#_ENREF_24)  (2012) | 8/16 | 50% |
| Ansah et al.[^25^](#_ENREF_25)  (2007) | 10/16 | 62.50% |
| Ayzenberg et al.[^10^](#_ENREF_10)  (2021) | 10/16 | 62.50% |
| Bilsel et al.[^36^](#_ENREF_36)  (2010) | 10/16 | 62.50% |
| Funakoshi et al.[^29^](#_ENREF_29)  (2018) | 15/24 | 62.50% |
| Iwasaki et al.[^26^](#_ENREF_26)  (2006) | 10/16 | 62.50% |
| Iwasaki et al.[^37^](#_ENREF_37)  (2009) | 10/16 | 62.50% |
| Lyons et al.[^38^](#_ENREF_38)  (2015) | 10/16 | 62.50% |
| Maniwa et al.[^18^](#_ENREF_18)  (2017) | 10/16 | 62.50% |
| Maruyama et al.[^19^](#_ENREF_19)  (2014) | 9/16 | 56.30% |
| Matsuura et al.[^14^](#_ENREF_14)  (2017) | 15/24 | 62.50% |
| Mihara et al.[^11^](#_ENREF_11)  (2010) | 10/16 | 62.50% |
| Mirzayan et al.[^27^](#_ENREF_27)  (2016) | 10/16 | 62.50% |
| Nishinaka et al.[^31^](#_ENREF_31)  (2014) | 7/16 | 43.80% |
| Ovesen et al.[^39^](#_ENREF_39)  (2011) | 10/16 | 62.50% |
| Pederzini et al.[^40^](#_ENREF_40)  (2017) | 10/16 | 62.50% |
| Sato et al.[^20^](#_ENREF_20)  (2018) | 11/16 | 68.80% |
| Shimada et al.[^22^](#_ENREF_22)  (2012) | 12/16 | 75% |
| Shimada et al.[^21^](#_ENREF_21)  (2005) | 8/16 | 50% |
| Tsuda et al.[^28^](#_ENREF_28)  (2005) | 9/16 | 56.30% |
| Ueda et al.[^15^](#_ENREF_15)  (2021) | 10/16 | 62.50% |
| Weigelt et al.[^23^](#_ENREF_23)  (2015) | 7/16 | 43.80% |
| Yamagami et al.[^16^](#_ENREF_16)  (2018) | 16/24 | 66.70% |
| Yamamoto et al.[^32^](#_ENREF_32)  (2006) | 10/16 | 62.50% |
